# Profiles of Immune Infiltration in Bladder Cancer and its Clinical Significance: an Integrative Genomic Analysis

**DOI:** 10.7150/ijms.42151

**Published:** 2020-03-05

**Authors:** Zonglong Wu, Kejia Zhu, Qinggang Liu, Yaxiao Liu, Lipeng Chen, Jianfeng Cui, Hongda Guo, Nan Zhou, Yaofeng Zhu, Yan Li, Benkang Shi

**Affiliations:** 1 Department of Urology, Qilu Hospital of Shandong University, Jinan, P.R. China.; 2 Key Laboratory of Urinary Precision Diagnosis and Treatment in Universities of Shandong, Jinan, P.R. China.

**Keywords:** bladder cancer, immune infiltration subtypes, The Cancer Genome Atlas, gene expression, CIBERSORT algorithm, personalized therapy

## Abstract

Tumor-infiltrating immune cells are closely related to the prognosis of bladder cancer. Analysis of tumor infiltrating immune cells is usually based on immunohistochemical analysis. Since many immune cell marker proteins are not specific for different immune cells, which may induce misleading or incomplete. CIBERSORT is an algorithm to estimate specific cell types in a mixed cell population using gene expression data. In this study, the CIBERSORT algorithm was used to identify the immune cell infiltration signatures. The gene expression profiles, mutation data, and clinical data were collected from The Cancer Genome Atlas (TCGA) database. Unsupervised consensus clustering was used to acquire the immune cell infiltration subtypes of bladder cancer based on the fractions of 22 immune cell types. Four immune cell clusters with different immune infiltrate and mutation characteristics were identified. In addition, this stratification has a prognostic relevance, with cluster 2 having the best outcome, cluster 1 the worst. These clusters showed distinct mRNA expression patterns. The characteristic genes in subtype cluster 1 were mainly involved in cell division, those in subtype cluster 2 were mainly related in antigen processing and presentation, those in subtype cluster 3 were mainly involved in epidermal cell differentiation, and those in subtype cluster 4 were mainly related in the humoral immune response. These differences may affect the development of the bladder cancer, the sensitivity to treatment as well as the prognosis. Through further validation, this study may contribute to the development of personalized therapy and precision medical treatments.

## Introduction

As one of the most common types of urological malignancies, bladder cancer (BLCA) remains a major global medical problem despite the availability of numerous new treatment options. Transitional cell (urothelial) carcinoma is responsible for 95% of BLCA cases 1. It is reported that there are 549,000 new cases of BLCA and 200,000 BLCA-related deaths per year in the world 2.

BLCA is highly heterogeneous on the genetic, expression, and histological 3. Accurate understanding of this heterogeneity can promote the molecular classification of BLCA and the management of personalized medicine. Numerous studies have reported the influence of the immune microenvironment on BLCA development and immunotherapy including intravesical bacillus Calmette-Guérin (BCG) and PD-1/PD-L1 blockade was long applied for the treatment of BLCA 4,5. The tumor microenvironment consists of immune cells, mesenchymal cells, endothelial cells, extracellular matrix (ECM) molecules, and inflammatory mediators 6. BLCA is an immunosensitive tumor which is infiltrated by tumor-infiltrating immune cells (TIICs) including T cells, macrophages, dendritic cells, neutrophils and mast cells 7-9. Studies have shown that the tumor microenvironment affects the gene expression of tumor tissues and the patient outcome, and therefore, has a diagnostic and prognostic value for BLCA 10-12. TIICs, which are main components of tumor microenvironment, have been reported closely related to the effectiveness of targeted drugs and clinical outcomes. However, most studies evaluated TIICs based on immunohistochemical analysis, which relies on a single marker to identify a specific immune cell 11-14. These traditional methods can be misleading and are not accurate as many marker proteins are not specific for different immune cells.

CIBERSORT is an algorithm to estimate specific cell types in a mixed cell population using gene expression data 15. In the present study, gene expression data was obtained from The Cancer Genome Atlas (TCGA) bladder urothelial cancer dataset and the fractions of 22 immune cell types were estimated by CIBERSORT. Four immune cell clusters with different clinical prognoses and mutation characteristics were identified by using unsupervised consensus clustering. It is hoped that this study may offer some important information for the understanding of the relationship between the heterogeneity of TIICs, and disease progression in BLCA, and provide insights into potential personalized therapeutic strategies for each subtype of BLCA.

## Materials and methods

### Database and genomic analysis

The mutation data, gene expression profiles, and clinical data of patients with BLCA were obtained from TCGA data portal (https://tcga-data.nci.nih.gov/tcga/). Gene expression data analysis was performed using the limma package of the R software. A fold change of > 2 and false discovery rate (FDR) of < 0.05 were used as cutoffs to identify differentially expressed genes (DEGs). The Maftools package was used to analyze and summarize the mutation data. Volcano plots and heat maps were generated using the ggplot2 and pheatmap packages, respectively.

### Evaluation of tumor-infiltrating immune cells

CIBERSORT algorithm was used to calculate the fractions of infiltrating immune cells. CIBERSORT is an analytical tool that estimates specific cell types in a mixed cell population using gene expression data; the algorithm was run using the 1000 permutations and LM22 signature 16. The fractions of immune cell were considered accurate when the CIBERSORT output reached p < 0.05.

### **Characteristic genes** enrichment analysis

The clusterProfiler package was used to perform GO enrichment analysis 17. An FDR of < 0.05 was used as the cut-off value.

### Statistical analysis

The unpaired t test was used to assess the difference between the immune fractions from tumor and non-tumor tissues. The median of the proportion of each cell type was computed for survival analysis, and survival curves were constructed by the Kaplan-Meier method and compared using the means obtained from the log rank test. To investigate whether distinct classes of tumor-infiltrating immune cells are present in different tumors, the patients were clustered into four groups based on the consensus proportion of each cell type. The differences of tumor mutation burden (TMB) in case of genes from each cluster were analyzed by one-way ANOVA, followed by Tukey's multiple-comparison post-hoc test. All analyses were performed using the R software (version 3.6).

## Results

### Composition and prognostic value of immune cells in bladder cancer

**Figure 1:**
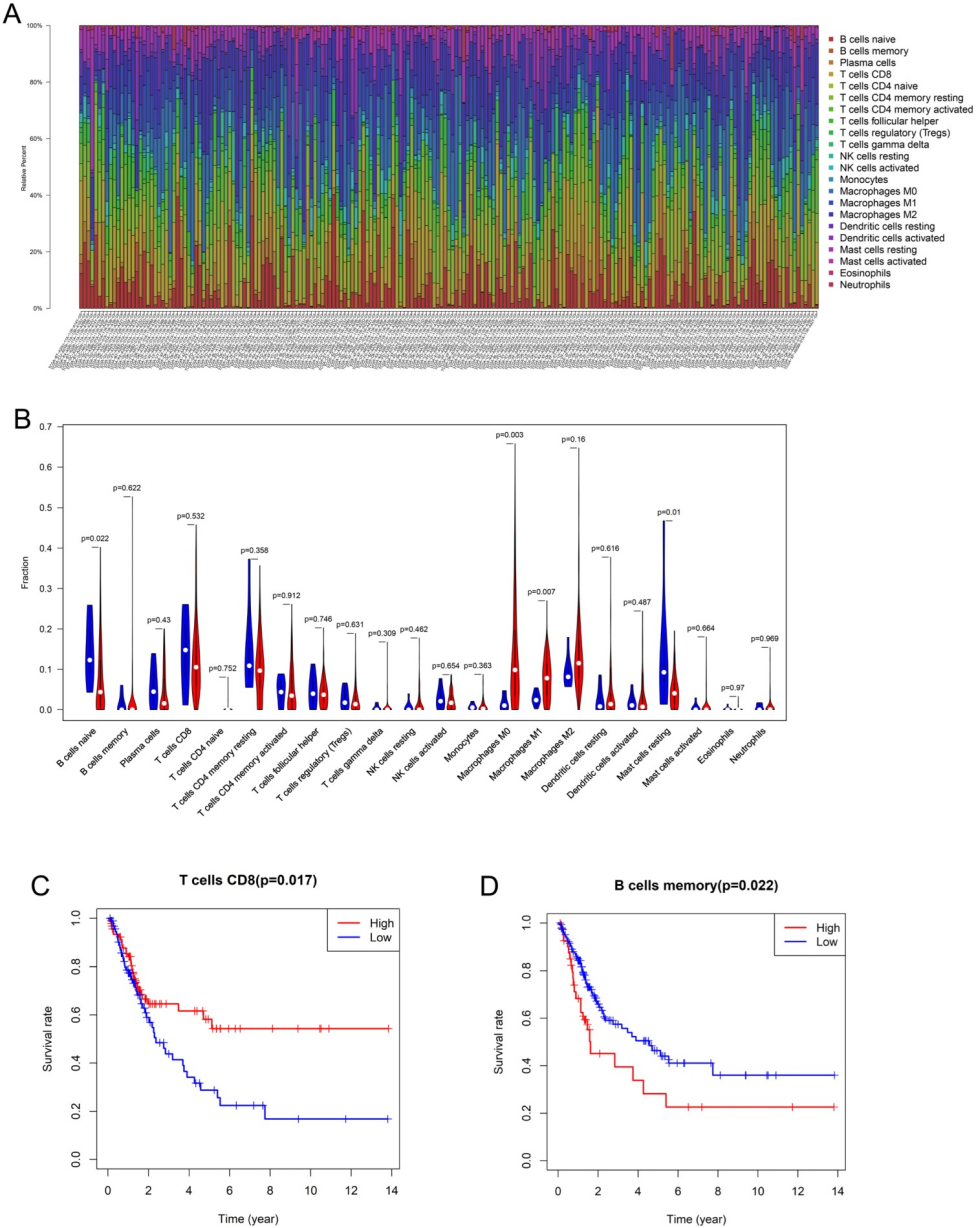
** Composition and prognostic value of immune cells in bladder cancer (A)** The percent of 22 types of fractions of tumor-infiltrating immune cell in bladder cancer. **(B)** 22 types of adaptive and innate immune cells in tumor and normal tissue groups. The fractions of M0 and M1 macrophages were consistently higher in the bladder cancer tissue than those of the normal tissue, whereas the fraction of naive B cells and resting mast cells was significantly lower in bladder cancer tissue (by unpaired t test). **(C, D)** The Kaplan-Meier survival curve of CD8 T cell and memory B cells in bladder cancer. Patients with high CD8 T cell fraction had a higher overall survival (HR 0.571, 95% CI 0.365-0.8932, p = 0.0149) whereas memory B cells (HR 1.765, 95% CI 0.9926-3.138, p = 0.0221) were associated with poor prognosis.

Using the CIBERSORT algorithm, 22 subpopulations of immune cells in 199 samples (192 tumor tissues, 7 normal tissues) were investigated (Figure 1A). The fractions of M0 and M1 macrophages were higher in the tumor tissues than in the normal tissues, whereas the fractions of naive B cells and resting mast cells were significantly lower in the tumor tissues (Figure 1B). M2 macrophages (13.7%) were the most abundant infiltrating immune cells in BLCA, followed by M0 macrophages (13.2%), CD8 T cells (12.4%), and CD4 T cells (10.2%). Then, Kaplan-Meier analysis and log-rank test were performed to analyze the prognostic value of the tumor-infiltrating immune cells. We found that CD8 T cells were associated with good prognosis (HR 0.571, 95% CI 0.365-0.8932, p = 0.0149), whereas memory B cells (HR 1.765, 95% CI 0.9926-3.138, p = 0.0221) were associated with poor prognosis in patients with BLCA (Figure 1C, D).

### Consensus clustering of immune cells identified four clusters of immune cell subtypes of BLCA

**Figure 2:**
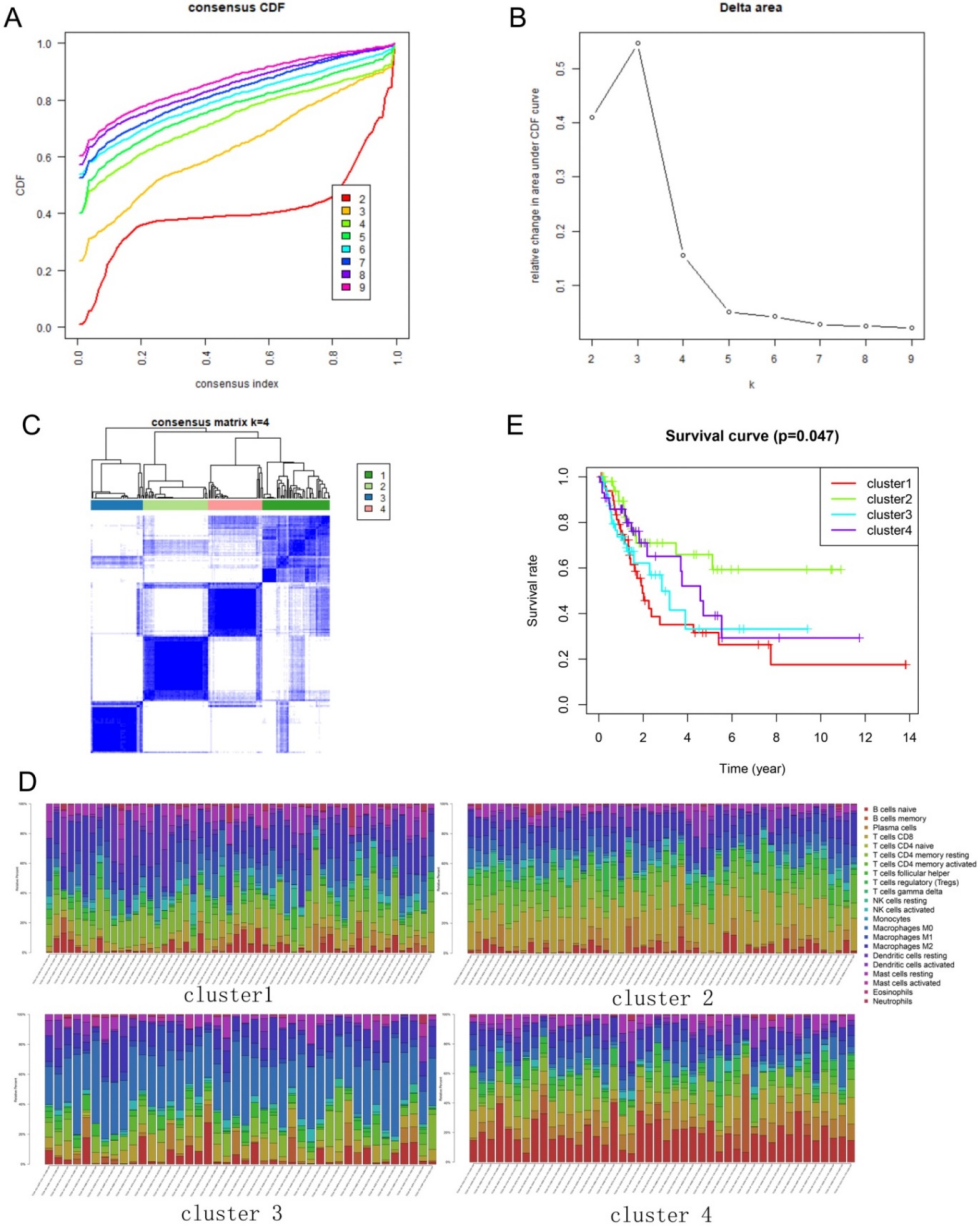
** Consensus clustering of immune cells identified four clusters of bladder cancer. (A)** Consensus clustering cumulative distribution function (CDF) for k = 2 to 9. **(B)** Relative change in area under CDF curve for k = 2 to 9. **(C)** Results of unsupervised consensus clustering identified four clusters. **(D)** The tumor-infiltrating immune cell proportions in four clusters. **(E)** The Kaplan-Meier survival curve of patients in different clusters.

The immune infiltration varies considerably at the individual level, and partially reflects the prognosis. Using unsupervised consensus clustering, we identified immune cell subtypes of BLCA with different clinical characters based on 22 different types of immune cells. Based on the similarity of immune infiltration, k = 4 was determined as the optimal number of clusters (Figure 2A, B). The consensus matrix heatmap revealed the four clusters that were identified (Figure 2C). The four clusters comprised different proportions of immune cells (Figure 2D). The fractions of the 22 immune cell types in each cluster are shown in Tables 1-4. Moreover, each cluster was associated with different clinical outcomes (Figure 2E). Significantly longer overall survival (OS) was found in the cluster 2 subgroup than that in other clusters.

### Identification of the expression profile features of genes from the four clusters

**Figure 3:**
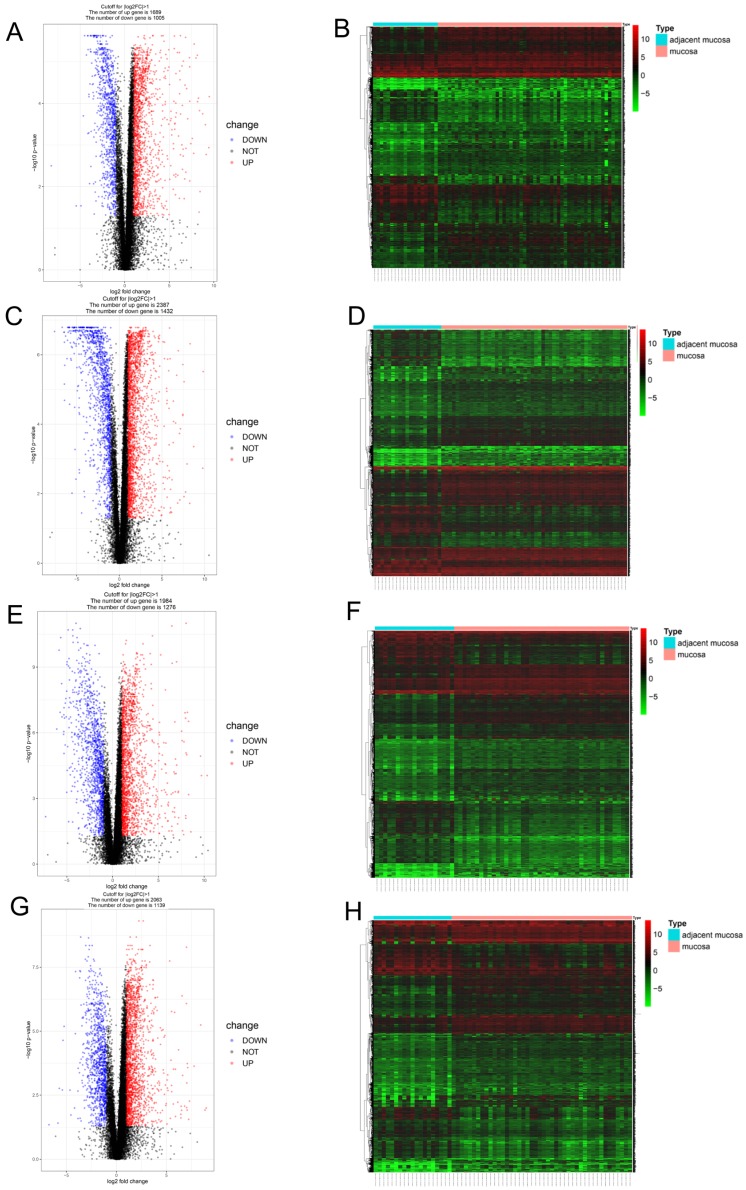
** Identification of gene expression profile feature in four clusters. (A, B)** The volcano plot and heatmap show the 2694 genes (1689 up-regulated and 1005 down-regulated) identified in cluster 1. **(C, D)** The volcano plot and heatmap show the 3819 genes (2387up-regulated and 1432 down-regulated) identified in cluster 2. **(E, F)** The volcano plot and heatmap show the 3260 genes (1984 up-regulated and 1276 down-regulated) identified in cluster 3. **(G, H)** The volcano plot and heatmap show the 3202 genes (2063 up-regulated and 1139 down-regulated) identified in cluster 4.

Since different clusters showed variations in the infiltrating immune cell types and patient outcomes, we explored the DEGs in the different clusters, compared to the genes in the normal tissues. In cluster 1, a total of 2694 DEGs (1689 upregulated and 1005 downregulated genes) were identified (Figure 3A) and visualized using a heatmap (Figure 3B). In cluster 2, a total of 3819 DEGs (2387 upregulated and 1432 downregulated genes) were identified (Figure 3C) and visualized using a heatmap (Figure 3D). In cluster 3, a total of 3260 DEGs (1984 upregulated and 1276 downregulated genes) were identified (Figure 3E) and visualized using a heatmap (Figure 3F). In cluster 4, a total of 3202 DEGs (2063 upregulated and 1139 downregulated genes) were identified (Figure 3G) and visualized using a heatmap (Figure 3H).

### Identification of characteristic genes in the four clusters and functional enrichment analysis

**Figure 4:**
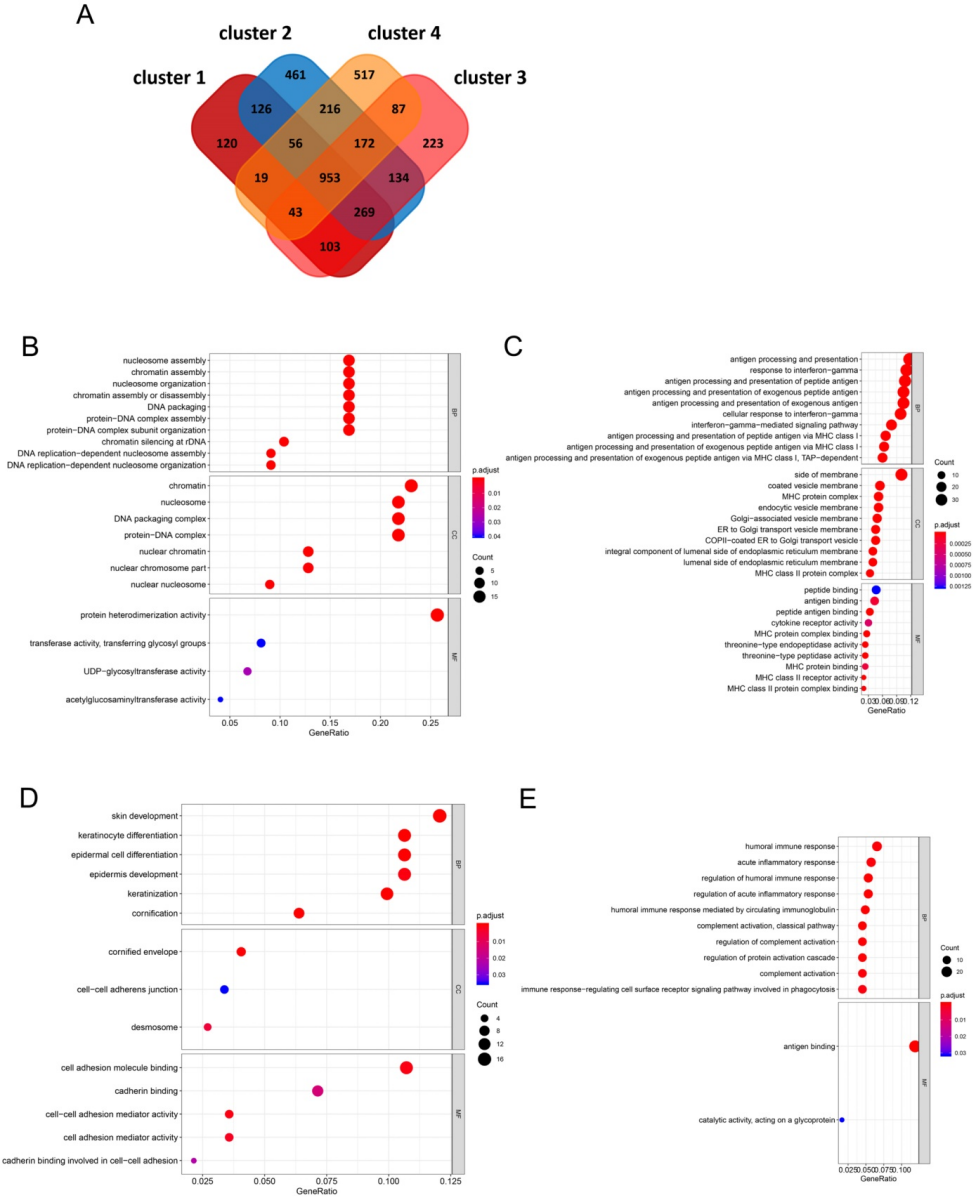
** Identification of characteristic genes of four clusters and functional enrichment analysis (A)** In the Venn diagrams, co-expression of upregulated and downregulated genes in four clusters. (B-E) The biological process, cellular component, and molecular function terms in four clusters.

A total of 953 DEGs were found to be overlapping in the four clusters. A total of 120, 461, 223, and 517 characteristic genes were identified in cluster 1, cluster 2, cluster 3, and cluster 4, respectively (Figure 4A). GO enrichment analysis of the characteristic genes in cluster 1 indicated that these genes were involved in “nucleosome assembly”, “chromatin assembly”, and “nucleosome organization” in the 'biological process' category. “Nucleosome” was the most enriched term in the 'cellular components' category, while “protein heterodimerization activity” was the most significantly enriched term in the 'molecular function' category (Figure 4B). In case of the genes from cluster 2, “antigen processing and presentation”, “response to interferon-gamma”, and “antigen processing and presentation of peptide antigen” were enriched in the 'biological process' category, “side of membrane”, “coated vesicle membrane”, and “MHC protein complex” were enriched in the 'cellular components' category, and “peptide binding” and “antigen binding” were enriched in the 'molecular function' category (Figure 4C). In case of genes from cluster 3, “keratinocyte differentiation” and “epidermal cell differentiation” were enriched in the 'biological process' category, “cornified envelope” was enriched in the 'cellular components' category, and “cell adhesion molecule binding” was enriched in the 'molecular function' category (Figure 4D). In case of genes from cluster 4, “humoral immune response”, “acute inflammatory response”, and “regulation of humoral immune response” were enriched in the 'biological process' category, no term was enriched in the 'cellular components' category, and “antigen binding and catalytic activity” and “acting on a glycoprotein” were enriched in the 'molecular function' category (Figure 4E).

### Identification of the mutation profile features of genes from each cluster

**Figure 5:**
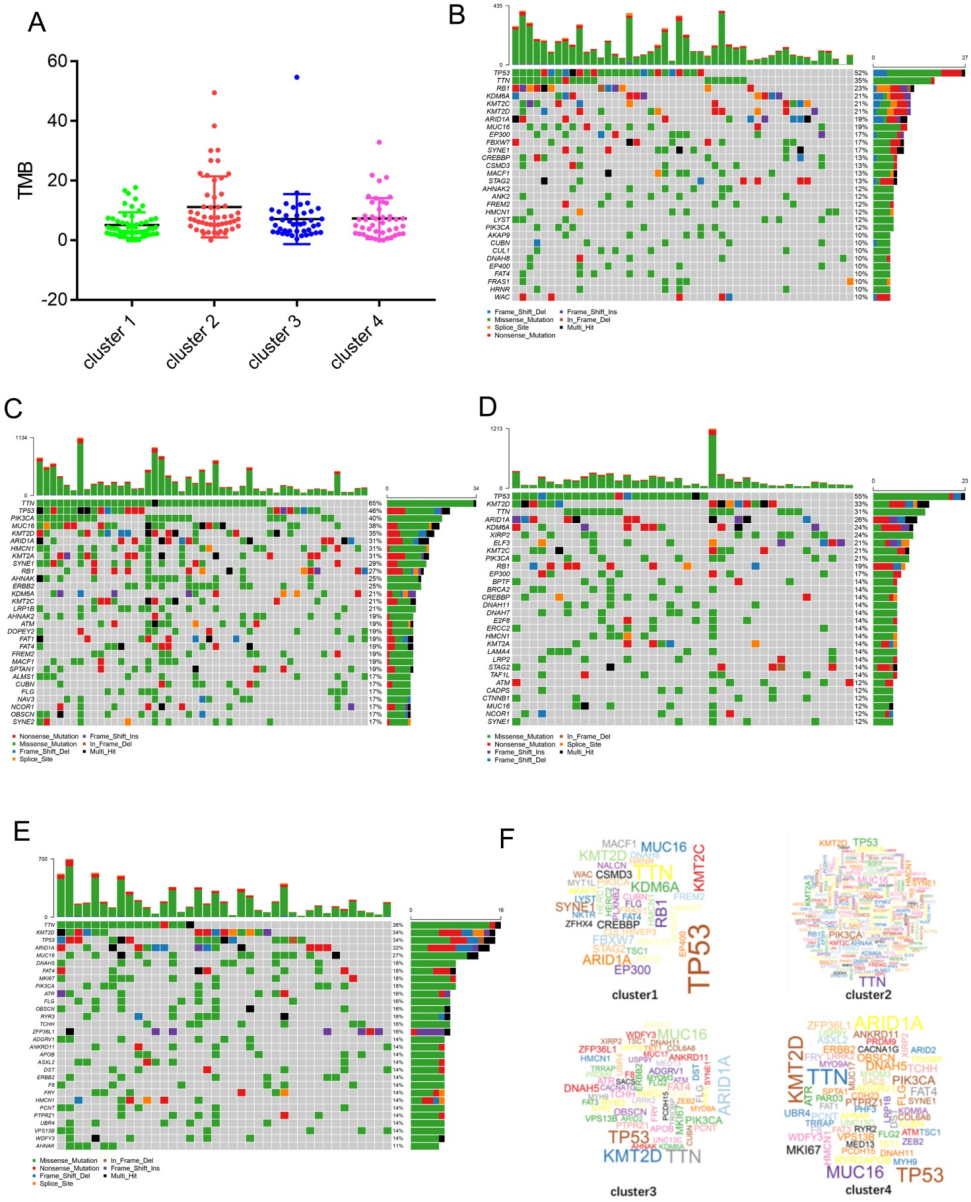
** Identification of mutation profile feature of each cluster. (A)** The TMB in four clusters. **(B-E)** The mutant genes and mutation profile of bladder cancer in four clusters. **(F)** Gene cloud map shows the name of mutant genes in four clusters. The size of gene names is proportional to the number of samples mutated for each gene.

It is well-known that cancer may results from the accumulation of somatic DNA mutations. High TMB leads to the formation of more new antigens, making tumors more immunogenic and more sensitive to immunotherapy. We downloaded the mutation data for each cluster from the TCGA database and calculated the respective TMB values. The results show that compared with the other clusters, cluster 2 had a higher TMB (Figure 5A). The alteration landscapes of the four clusters are shown in Figure 5B-E. In cluster 1, 6 genes were mutated by > 20%: TP53 (52%), TTN (35%), RB1(23%), KDM6A (21%), KMT2C (21%), and KMT2D (21%). In cluster 2, 15 genes were mutated by > 20%: TTN (65%), TP53 (46%), PIK3CA (40%), MUC16 (38%), KMT2D (35%), ARID1A (31%), HMCN1 (31%), KMT2A (31%), SYNE1 (29%), RB1 (27%), AHNAK (25%), ERBB2 (25%), KDM6A (21%), KMT2C (21%), and LRP1B (21%). In cluster 3, 9 genes were mutated by > 20%: TP53 (55%), KMT2D (33%), TTN (31%), ARID1A (26%), KDM6A (24%), XIRP2 (24%), ELF3 (21%), KMT2C (21%), and PIK3CA (21%). In cluster 4, 5 genes were mutated by > 20%: TTN (36%), KMT2D (34%), TP53 (34%), ARID1A (32%), and MUC16 (27%). There were fewer mutant genes in cluster 1, while cluster 2 had the greatest number of mutated genes (Figure 5F).

## Discussion

While the introduction of novel targeted drugs can increase treatment options of BLCA, these treatments are only effective in certain BLCA patients. The identification of subtypes of BLCA will help biological research and personalized treatment of BLCA. TCGA is an open-access database that uses a genome-wide approach to reveal the genetic characteristics of cancers. Many studies on cancers such as BLCA have screened diagnostic and prognostic biomarkers using TCGA 18-20. Patient subgroups with different treatment response and prognosis can also be identified using TCGA.

In the present study, four immune clusters for BLCA were identified and these four clusters showed different patterns of infiltrating immune cell gene expression signatures and mutation characteristics. Among all clusters, cluster 1 was associated with the worst prognosis, and cluster 2, with the best outcome. Cluster 1 was characterized by the increased infiltration of M2 macrophages and CD4 memory T cells. M2 macrophages play a role in anti-inflammatory processes, tissue repair and remodeling, the immune regulation process, parasite clearance, and the tumor promotion process 21. The immunosuppressive factors released by M2 macrophages may support immune evasion in bladder cancer. Tissue resident memory T cells are a key factor in making tumors dormant; hence, it is essential to establish a cancer-immune system balance 22. The feature of cluster 2 was the increased infiltration of CD8 T cells. Studies have shown that the accumulation of CD8 T lymphocytes in tumors often indicates good clinical outcomes 23. Under hypoxic conditions, CD8 T cells can differentiate into lytic effector cells, increase the expression of interferon gamma (IFNγ), Fas ligand (FASL), granule B (GZMB), and inhibit tumor cell proliferation 24,25. M0 macrophages were the most abundant infiltrating immune cells in case of cluster 3, and naive B cells were the most abundant infiltrating immune cells in case of cluster 4. Previous studies have shown that activated naive B cells are required for the initiation of T cell immune responses, owing to their co-stimulatory activity and ability to produce cytokines for the activation and expansion of effector and memory TH cell populations 26,27.

The four subtypes also showed different gene expression signatures and were associated with different biological processes. The signature genes in cluster 1 were mainly involved in the process of cell division. The signature genes in cluster 2 were mainly involved in the response to interferon-gamma and antigen processing and presentation. The signature genes in cluster 3 were mainly involved in epidermal cell differentiation. The signature genes in cluster 4 were mainly involved in the humoral immune response. This result reflects a profound link between gene expression and immune cell infiltration. Cancer is a genetic disease and caused by the accumulation of somatic mutations 28. In a sense, the characteristics of immune infiltration also represent the genetic characteristics of cancer. Next, we analyzed the mutation characteristics of genes from each cluster. The results showed that the mutant genes and mutation frequencies in each cluster were different. In cluster 1, 6 genes were mutated by > 20%: TP53 (52%), TTN (35%), RB1 (23%), KDM6A (21%), KMT2C (21%), and KMT2D (21%). In cluster 2, 15 genes were mutated by > 20%: TTN (65%), TP53 (46%), PIK3CA (40%), MUC16 (38%), KMT2D (35%), ARID1A (31%), HMCN1 (31%), KMT2A (31%), SYNE1 (29%), RB1 (27%), AHNAK (25%), ERBB2 (25%), KDM6A (21%), KMT2C (21%), and LRP1B (21%). In cluster 3, 9 genes were mutated by > 20%: TP53 (55%), KMT2D (33%), TTN (31%), ARID1A (26%), KDM6A (24%), XIRP2 (24%), ELF3 (21%), KMT2C (21%), and PIK3CA (21%). In cluster 4, 5 genes were mutated by > 20%: TTN (36%), KMT2D (34%), TP53 (34%), ARID1A (32%), and MUC16 (27%).

We found that TP53 and TTN are the major mutant genes in cluster 1. The tumor suppressor gene TP53 has been reported to be mutated in more than 50% of human malignancies, and thus, promote the development and progression of cancer 29. In comparison with other groups cluster 2 have more mutated gene and a higher TMB. It has been observed in other tumors that high TMB may reflects the presence of new antigens, thereby increasing lymphocyte infiltration in the tumor microenvironment 30,31. The cluster 3 had similar mutation characteristics with cluster 1; TP53 and TTN were the major mutant genes. Additionally, we found that the mutation in TP53 in case of cluster 4 is significantly lower than that in case of the other clusters. Furthermore, there was no dominant mutant gene in cluster 4. TP53 mutations often result in unstable tumor genomes and impaired DNA repair capacity, therefore; TP53 mutant tumors may be more sensitive to DNA damage factors 3. Conversely, a wild-type TP53 gene expression signature in BLCA have been shown to be resistant to neoadjuvant chemotherapy 32, so the prognosis of patients in this cluster 4 may likely to be resistant to neoadjuvant chemotherapy. BLCA is one of the most immunotherapy-responsive solid tumors. High TMB is associated with the response to immune checkpoint inhibitors (ICIs) in BLCA 33. Patients in cluster 2 have high infiltration of CD8 T cells. Moreover, high levels of TMB suggest that these patients may benefit from ICIs, such as anti‐PD‐1 or anti‐CTLA‐4 therapies. Cluster 3 was highly expressed basal and squamous differentiation markers. Basal bladder cancer originates from basal cells and stem cells of the bladder urothelium 34, which characterized by enrichment of squamous, stemness and EMT markers 35. This subtype may be more sensitive to neoadjuvant chemotherapy. In basal BLCA, epidermal growth factor receptor (EGFR) pathway was usually activated 36. Therefore, anti-EGFR therapy may provide benefits for patients of this subtype.

In conclusion, based on immune cell types, we used unsupervised consensus clustering to identify four subtypes of BLCA. These four immune subtypes showed distinct mRNA expression patterns and different mutation characteristics. These differences may affect the development of the tumors and the sensitivity towards the treatment. We also provided a potential treatment strategy for different subtypes. This study may be helpful for the exploration of personalized treatments.

## Figures and Tables

**Table 1:** The fractions of tumor-infiltrating immune cells in cluster 1

Immune cells in cluster 1	Fraction
Macrophages M2	0.199609146
T cells CD4 memory resting	0.162765006
Macrophages M0	0.093887564
Macrophages M1	0.073074785
T cells CD8	0.071416282
Dendritic cells resting	0.060103665
Mast cells resting	0.05985775
B cells naive	0.038816143
Dendritic cells activated	0.037608841
T cells follicular helper	0.029186935
T cells regulatory (Tregs)	0.028161336
T cells CD4 memory activated	0.02725125
NK cells activated	0.025787568
Plasma cells	0.020464493
Mast cells activated	0.01548266
Monocytes	0.015375007
NK cells resting	0.014180805
Neutrophils	0.013552612
B cells memory	0.009292636
Eosinophils	0.002728938
T cells gamma delta	0.001396577
T cells CD4 naive	0

**Table 2:** The fractions of tumor-infiltrating immune cells in cluster 2

Immune cells in cluster 2	Fraction
T cells CD8	0.233496
Macrophages M2	0.120392
T cells CD4 memory activated	0.112792
Macrophages M1	0.096799
T cells CD4 memory resting	0.066103
Macrophages M0	0.05563
T cells follicular helper	0.054722
Plasma cells	0.041017
Mast cells resting	0.035316
B cells naive	0.03482
Dendritic cells activated	0.027455
Dendritic cells resting	0.027433
NK cells resting	0.025294
T cells regulatory (Tregs)	0.020309
NK cells activated	0.019001
Monocytes	0.010053
B cells memory	0.005778
Neutrophils	0.005571
T cells gamma delta	0.004365
Mast cells activated	0.002259
Eosinophils	0.001397
T cells CD4 naive	0

**Table 3:** The fractions of tumor-infiltrating immune cells in cluster 3

Immune cells in cluster 3	Fraction
Macrophages M0	0.335242
Macrophages M2	0.124094
T cells CD4 memory resting	0.07798
T cells CD8	0.074366
Macrophages M1	0.070703
B cells naive	0.053255
Mast cells resting	0.040685
T cells follicular helper	0.040265
Plasma cells	0.030809
T cells CD4 memory activated	0.030375
Dendritic cells resting	0.024294
NK cells activated	0.021609
Dendritic cells activated	0.019461
T cells regulatory (Tregs)	0.011788
Mast cells activated	0.011171
B cells memory	0.010108
NK cells resting	0.009934
Neutrophils	0.004579
T cells gamma delta	0.003108
T cells CD4 naive	0.002469
Monocytes	0.002026
Eosinophils	0.00168

**Table 4:** The fractions of tumor-infiltrating immune cells in cluster 4

Immune cells in cluster 4	Fraction
B cells naive	0.194482
T cells CD8	0.107602
T cells CD4 memory resting	0.093953
Plasma cells	0.09387
Macrophages M2	0.093446
Macrophages M0	0.074231
Macrophages M1	0.067259
Mast cells resting	0.05007
T cells follicular helper	0.040493
T cells CD4 memory activated	0.035873
T cells regulatory (Tregs)	0.0339
Dendritic cells activated	0.032261
B cells memory	0.021131
NK cells activated	0.020878
Dendritic cells resting	0.01669
Monocytes	0.00695
T cells gamma delta	0.005126
Neutrophils	0.004548
NK cells resting	0.003773
Mast cells activated	0.003042
Eosinophils	0.000312
T cells CD4 naive	0.00011
